# Editorial: The role of regulatory networks in virulence and antimicrobial resistance of microbial pathogens

**DOI:** 10.3389/fmicb.2024.1370093

**Published:** 2024-02-12

**Authors:** Abdelaziz Elgaml, Rami Elshazli, Shin-ichi Miyoshi

**Affiliations:** ^1^Microbiology and Immunology Department, Faculty of Pharmacy, Mansoura University, Mansoura, Egypt; ^2^Microbiology and Immunology Department, Faculty of Pharmacy, Horus University - Egypt, New Damietta, Egypt; ^3^Biochemistry and Molecular Genetics Department, Faculty of Physical Therapy, Horus University - Egypt, New Damietta, Egypt; ^4^Graduate School of Medicine, Dentistry and Pharmaceutical Sciences, Okayama University, Okayama, Japan

**Keywords:** antimicrobial resistance, global regulators, microbial pathogens, regulatory networks, virulence factors

Antimicrobial resistance (AMR), commonly referred to as microbial drug resistance, is a serious issue for world health. It describes the capacity of microorganisms to fend off the effects of medications that were once successful in curing infections that they cause (Adefisoye and Olaniran, 2023). Numerous research areas are progressing within this outline, including the identification of AMR and its spread mechanisms, innovation of novel possible therapeutic approaches, enhanced epidemiological investigation of pathogens and their AMR and creation of novel diagnostic tackles. Therefore, the primary aim of this Research Topic was to compile current advancements in the aforementioned domains, resulting in a set of 6 contributions.

Drug inactivation, drug target alteration, drug uptake limitation and drug active efflux are the four primary mechanisms of microbial resistance (Reygaert, 2018). In their review article, Li et al. gave a concise summary of the specific mechanisms of resistance of *Klebsiella pneumonia* to carbapenems, ceftazidime/avibactam, tigecycline and colistin. They illustrated that the acquisition of a plasmid containing *bla*_KPC_ and *bla*_NDM_ genes, mutations in *bla*_KPC_ and porin genes (*ompK35* and *ompK36*) and overexpression of *bla*_KPC_ usually cause carbapenem and ceftazidime/avibactam resistance in living organisms. Adaptive evolution of tigecycline resistance can occur by efflux pump overexpression, *tet (A)* variant plasmid acquisition and ribosomal protein changes. Certain genetic changes in chromosomes replace lipid A's phosphate groups with cationic compounds resulting in colistin resistance. One key point of this review is that the generation of resistance mutants is influenced by the internal environment and the pressure exerted by antibiotic selection that may convert antibiotic-susceptible strains to resistant. The authors explained that the internal milieu within the human host could potentially act as a significant reservoir of *K. pneumoniae* strains that are resistant to treatment. The emergence of resistant strains could be significantly diminished by inhibiting this conversion process. Moreover, in their original article, Nageeb et al. exhibited an exhaustive epidemiological and statistical examination of potential membrane proteins and efflux-pump variants associated with carbapenem susceptibility in *Acinetobacter baumannii*, including an important efflux-pump structural protein encoded by *adeB* gene. This research illuminates the clinical utility of these elements as diagnostic markers and treatment modification targets, thereby paving the way for more targeted investigations of candidate components. In addition, in her review article, Chetri provided a comprehensive analysis and extensive overview of several efflux pump families, along with a detailed discussion of their possible uses. In addition, this review examined the diverse biological functions of efflux pumps, such as their involvement in biofilm formation, quorum sensing (QS), bacterial survival and virulence. Furthermore, the study explored the genes and proteins associated with efflux pumps and their potential implications for antimicrobial resistance and the detection of antibiotic residues. The concluding discourse revolved with efflux pump inhibitors, namely those originating from botanical sources.

The intricate behavior of microbial pathogen communities, including virulence and drug resistance, is primarily controlled by regulatory networks. Crucially, these networks' regulatory molecules have the ability to impact other pathogens as well as the host (Gelfand, 2006; Cho et al., 2007). The microbial pathogen regulatory networks include the transcription factors, the two-component systems, QS and short RNA molecules known as small non-coding RNAs (sRNAs) (Dietrich et al., 2013; Abdel-Sattar et al., 2016; Groisman, 2016; Quendera et al., 2020). Target genes and downstream regulatory proteins are expressed under the control of master regulators in these networks. They enable pathogens to adapt to a variety of environmental circumstances and enhance their virulence and survival tactics. In their original article, Fu et al. described the role of the Crp/Fnr family transcriptional regulator named ArcR in resistance to fluoroquinolones in *Staphylococcus aureus*. They demonstrated that the absence of *arcR* diminished the ability of *S. aureus* to withstand fluoroquinolone drugs, mostly due to a deficiency in its ability to respond to oxidative stress. This study depicted that *arcR* mutation results in a decrease in the expression of the primary catalase gene *katA* through control of its promoter region, while the overexpression of *katA* reinstated the bacterium's resistance to oxidative stress and antibiotics. Therefore, this study has proven the role of ArcR in enhancing bacterial resistance to oxidative stress and, consequently, to fluoroquinolone antibiotics. This opens the door for the innovation of new therapeutic targets via the involvement of the Crp/Fnr family to increase bacterial vulnerability to antibiotics.

The issue of microbial drug resistance is a result of multiple factors. The absence of new antimicrobial medication development is an important factor. The rate at which microorganisms become resistant has increased in recent decades, but the discovery and development of new drugs has slowed down significantly. The issue is made worse by the disparity between the rise of drug-resistant strains and the accessibility of efficient remedies (Kono et al., 2021). The primary objective of the study conducted by Yousef et al. was to identify a viable antibacterial substance from a range of plant extracts and oils, with the aim of impeding the growth of detrimental bacteria. This study showed that the extracts of *Geranium gruinum, Datura stramonium* and *Mentha spicata* had the highest efficacy as antibacterials in inhibiting the growth of *Bacillus thermophilus*. Conversely, *Mentha spicata* and *Ocimum bacilicum* were found to be the most effective oils in suppressing the growth of *B. thermophilus*. The findings of this study suggest that utilizing antimicrobial botanical extracts and oils could be used as novel therapeutic approaches for treatment of resistant pathogens. Moreover, the review article conducted by Saeed et al. explained in detail potential innovative therapeutic strategies for addressing multidrug resistant microorganisms, including CRISPR-based antimicrobials, bacteriophages, adjuvanted subunit vaccine, immunotherapies, live attenuated vaccines, DNA vaccines, nanomedicine, artificial intelligence, conjugated vaccines, shRNAs, siRNAs and virus-like particles.

Overall, it is clear that the pathophysiology of infectious diseases and, to some extent, the trajectory of infectious diseases are significantly influenced by the regulatory networks of microbial pathogens. Comprehending these regulatory networks would facilitate the development of more effective intervention strategies aimed at reducing pathogen-induced host damage as well as morbidity and mortality rates. To better understand microbial pathogenesis and develop new therapeutic approaches the primary goal of this Research Topic “*The role of regulatory networks in virulence and antimicrobial resistance of microbial pathogens”* was to compile novel concepts in the field of microbial drug resistance and regulatory network systems of microbial pathogens.

## Author contributions

AE: Writing – original draft, Writing – review & editing. RE: Writing – review & editing. S-iM: Writing – review & editing.
